# Dataset of plant composition change over seven years at the Zumwalt Prairie Preserve, Oregon, USA

**DOI:** 10.1016/j.dib.2019.105104

**Published:** 2020-01-07

**Authors:** Joshua P. Averett, Lesley R. Morris, Bryan A. Endress

**Affiliations:** a Eastern Oregon Agricultural Research Center, Oregon State University, Union, OR 97883, USA; b Eastern Oregon Agriculture and Natural Resource Program, Department of Animal and Rangeland Sciences, Oregon State University, One University Blvd, La Grande, OR, 97850, USA

**Keywords:** *Ventenata dubia*, Annual grass invasion, Grassland change, Pacific northwest Bunchgrass Prairie

## Abstract

The data and analyses presented here were collected at the Zumwalt Prairie Preserve (ZPP), northeastern Oregon. Vegetation composition was measured within 124 (1-ha) plots using the line point intercept method [1,2]. These data include vascular plant species abundance matrices at two different time periods, seven years apart (2008/2009 & 2015/2016); boxplots of species abundance (cover and frequency) change over time; Non-parametric Multiplicative Regression (NPMR) estimated abundance of *Ventenata dubia*, an invading non-native annual grass, in geographic and ordination (Non-metric Multidimensional Scaling ordination; NMS) space over time.

**Table:** Specifications Table

Subject	Biology
Specific subject area	Grassland vegetation community composition change
Type of data	Figures, Tables (csv)
How data were acquired	Repeat vegetation sampling within permanent plots
Data format	Analyzed, Raw
Parameters for data collection	Plant species foliar cover and frequency of occurrence; plant community type; vascular plant composition; nativity of plant species
Description of data collection	Plots (n = 124; area ∼ 1ha) were located using a stratified random design across ∼ 5100 ha. Line point intercept transects were established at each plot. Foliar cover of vascular plant species along transects and frequency of occurrence within quadrats that were aligned with transects were measured at two sample periods, seven years apart (summers of 2008/2009 and 2015/2016) to track vegetation change.
Data source location	Zumwalt Prairie Preserve, Oregon, USA. (45˚58′ N, 116° 97′ W)
Data accessibility	The data are available with this article
Related research article	Averett, J.P., Morris, L.R., Naylor, B. J., Taylor, R. V., Endress, B. A. Vegetation change over seven years in the largest protected Pacific Northwest Bunchgrass Prairie remnant. (in Press) PLoS ONE. doi.org/10.1371/journal.pone.0227337

**Table:** 

**Value of the data** • These data provide vegetation community change and plant invasion information for a threatened and understudied vegetation type of high conservation value (Pacific Northwest Bunchgrass Prairie; PNB). • These data can be used by researchers and land managers to inform conservation efforts and understand plant invasion/community dynamics in the PNB. • These data provide vegetation distribution/composition data for a PNB ecosystem early in the stages of invasion by an exotic annual grass and can be compared to future repeat-measurements to reveal impacts and dynamics of plant invasion in the PNB.

## 1 Data

**Fig. 1:**
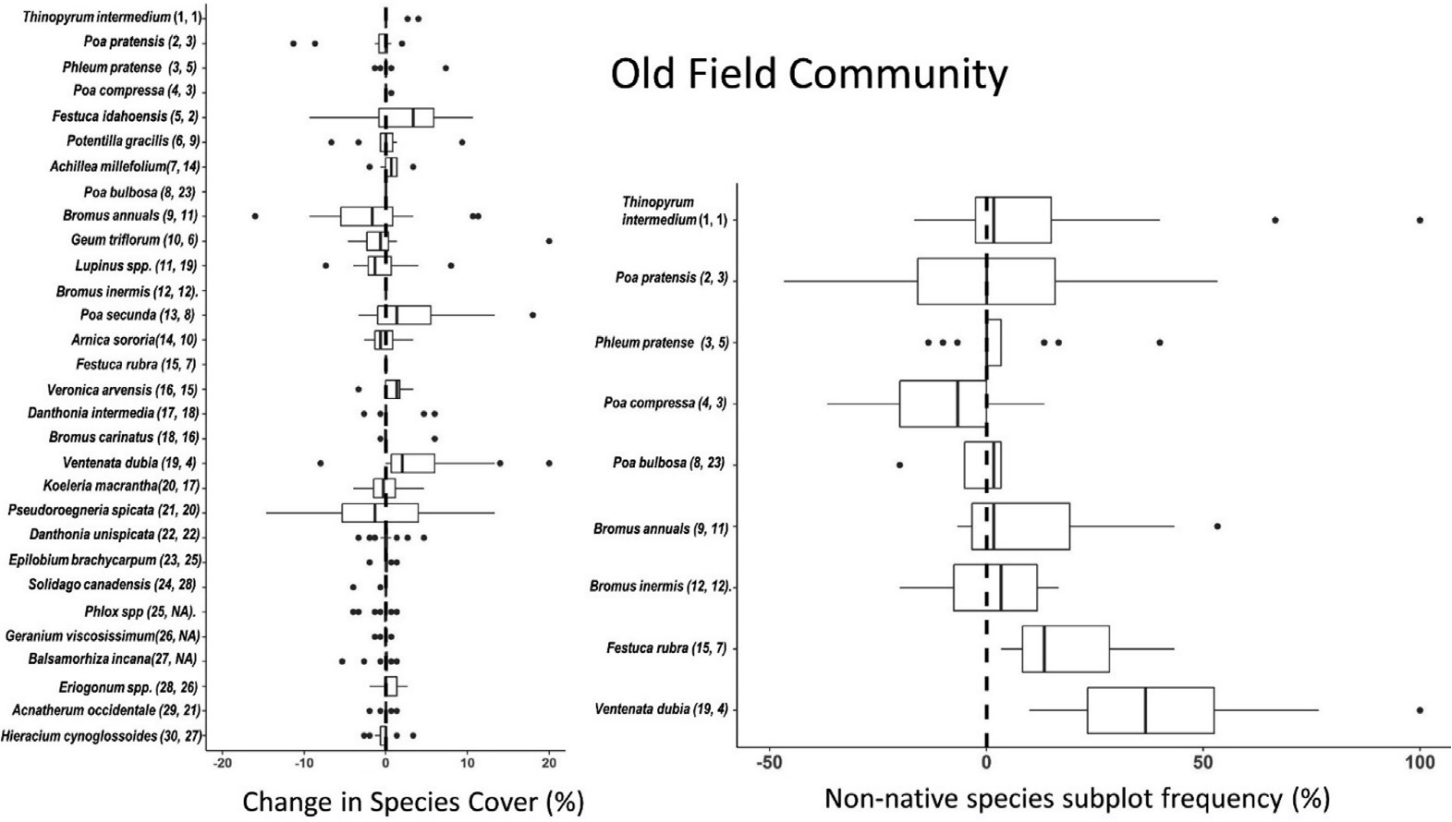
Left panel - Boxplots showing distributions of pairwise differences (plots are sample units) in percent foliar cover for the 30 dominant vascular plant species in the old field community (see Refs. [3,4] for identification and description of community types). Right panel – pairwise differences in frequency of occurrence within quadrats for the dominant non-native grass species in the old field community. Species are ordered by rank abundance (top to bottom) for the first sampling period (2008, 2009). Rank abundance values for each species and metric (foliar cover/frequency) during sampling period 1 (left) and sampling period 2 (right) are shown parenthetically.

**Fig. 2:**
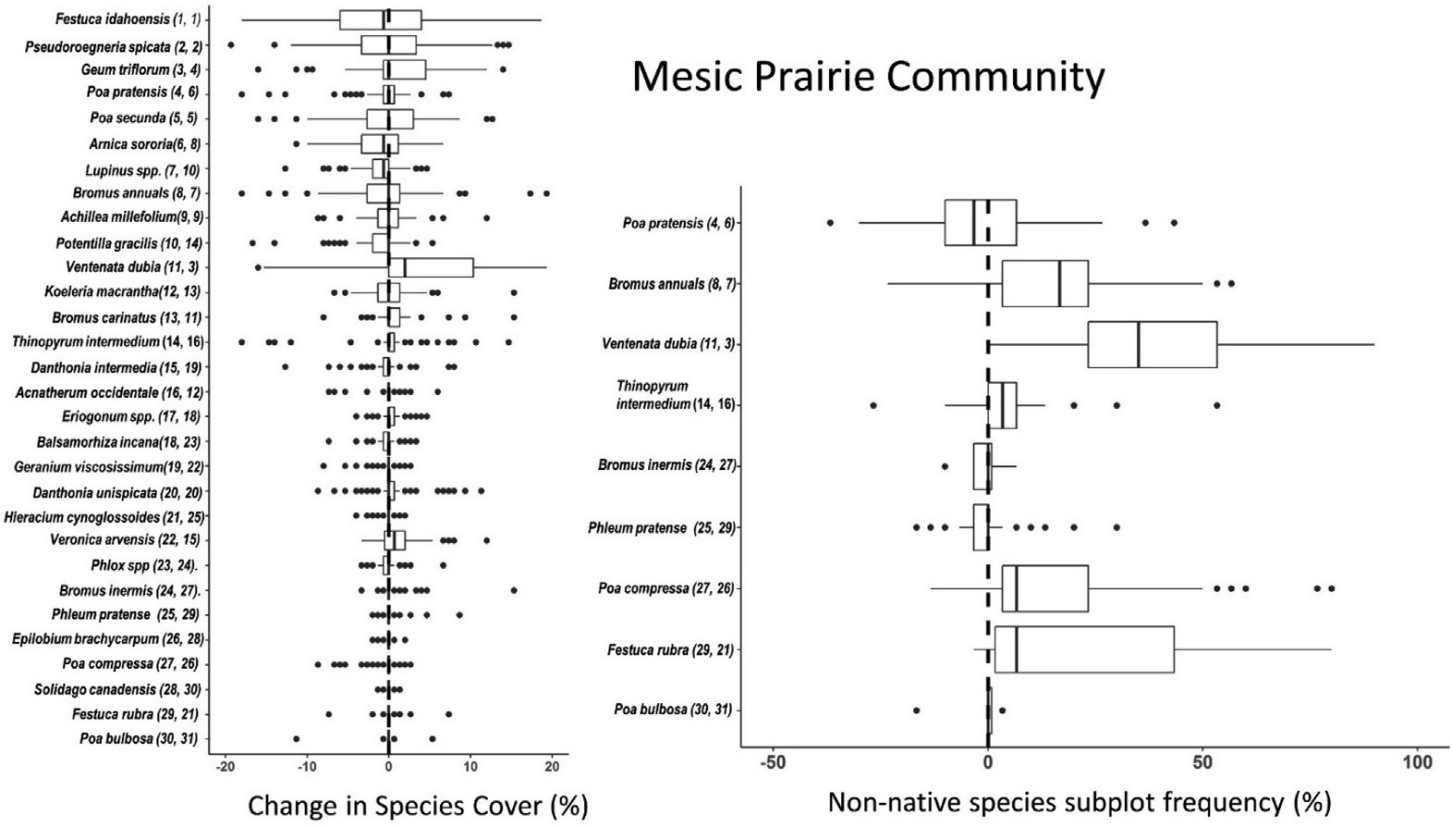
Left panel - Boxplots showing distributions of pairwise differences (plots are sample units) in percent foliar cover for the 30 dominant vascular plant species in the mesic prairie community. Right panel – pairwise differences in frequency of occurrence within quadrats for the dominant non-native grass species in the mesic prairie community. Species are ordered by rank abundance (top to bottom) for the first sampling period (2008, 2009). Rank abundance values for each species and metric (foliar cover/frequency) during sampling period 1 (left) and sampling period 2 (right) are shown parenthetically.

**Fig. 3:**
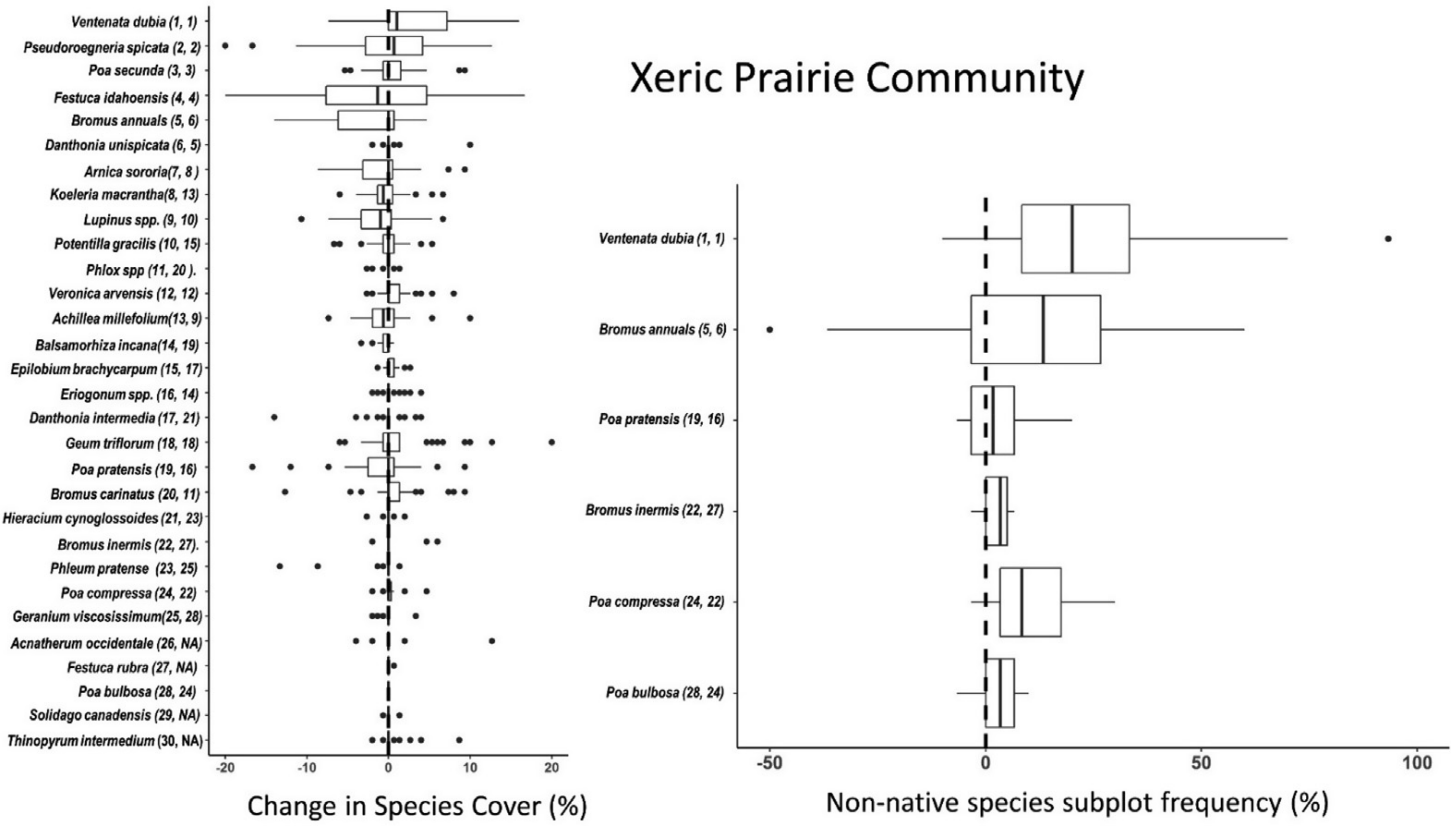
Left panel - Boxplots showing distributions of pairwise differences (plots are sample units) in percent foliar cover for the 30 dominant vascular plant species in the xeric prairie community. Right panel – pairwise differences in frequency of occurrence within quadrats for the dominant non-native grass species in the xeric prairie community. Species are ordered by rank abundance (top to bottom) for the first sampling period (2008, 2009). Rank abundance values for each species and metric (foliar cover/frequency) during sampling period 1 (left) and sampling period 2 (right) are shown parenthetically.

**Fig. 4:**
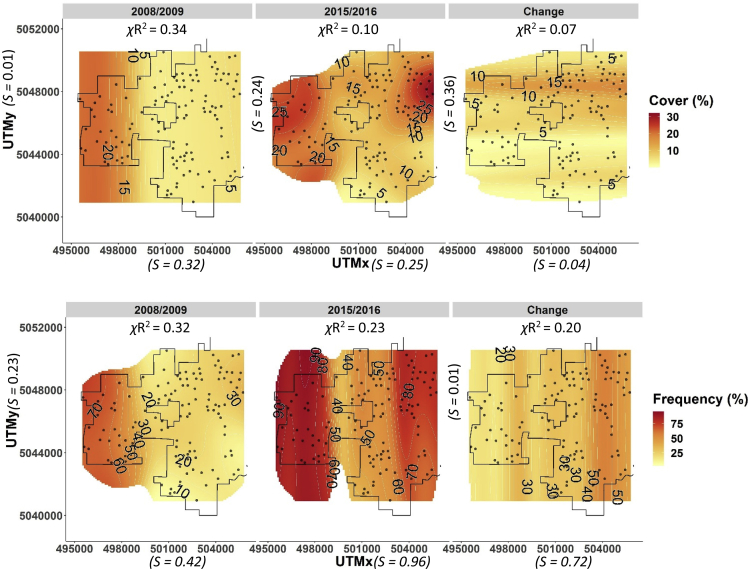
NPMR generated contour plots of percent foliar cover (top 3 panels), and frequency of occurrence within quadrats (bottom 3 panels) for both sampling periods as well as difference between sampling periods (change) for *Ventenata dubia* across the Zumwalt study area. Darker red shading indicates higher abundance or frequency and more change, and lighter yellow shading indicates lower abundance and change. Points are plots (sample units). Cross-validated R^2^ values are shown above figures and predictor sensitivity is depicted parenthetically.

**Fig. 5:**
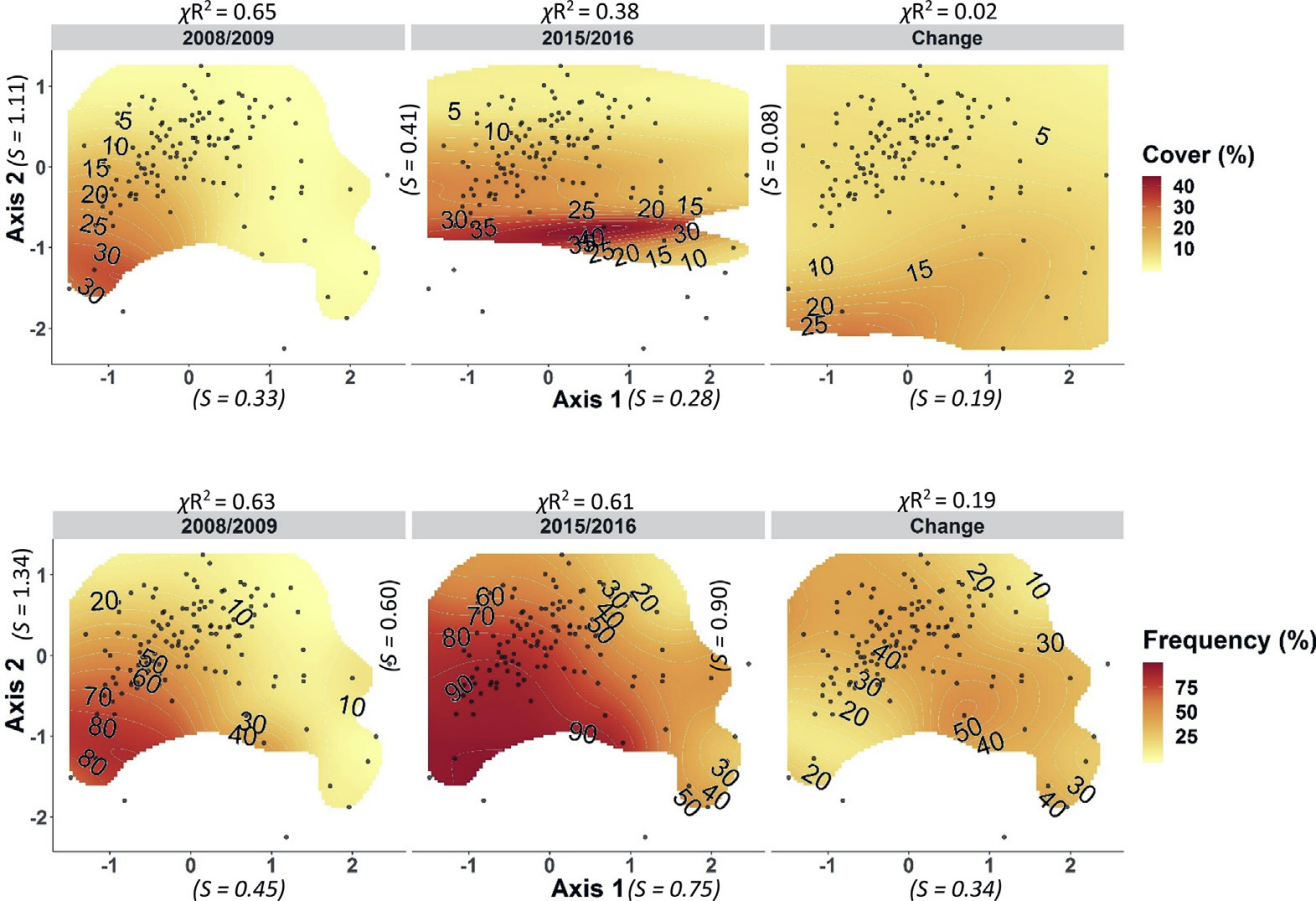
NPMR generated contour plots of percent foliar cover (top 3 panels), and frequency of occurrence within quadrats (bottom 3 panels) for both sampling periods as well as difference between sampling periods (change) for *Ventenata dubia* in NMS ordination space (predictors = NMS ordination axes 1 & 2). Darker red shading indicates higher abundance or more change, and lighter yellow shading indicates lower abundance or frequency and change. Points are plots (sample units). Cross-validated R^2^ values are shown above figures and predictor sensitivity is depicted parenthetically.

These data and analyses support the research article “Vegetation change over seven years in the largest protected Pacific Northwest Bunchgrass Prairie remnant” Averett et al. (in Press) [3]. The presented data include: (1) Downloadable CSV files (Appendix A) that include species abundance matrices by plot and community type (communities defined in Ref. [4]) for two sampling periods (sample period 1 = summers of 2008 & 2009; sample period 2 = summers of 2015/2016), seven years apart in the ZPP, and (Appendix A) that includes a vascular plant species list of the 31 dominant species in our dataset including species scientific name, common name, family, life form (forb or grass), life span (annual or perennial), and nativity (native or not to contiguous United States); (2) Boxplots showing change in percent foliar cover of dominant (n = 30) species and change in frequency of quadrats occupied per plot by dominant non-native grass species over time and stratified by grassland community type Fig. 1, Fig. 2, Fig. 3); and (3) NPMR generated contour plots of *Ventenata dubia* (the most dominant non-native plant species at the ZPP) abundance as a function of both geographic and NMS ordination space (Fig. 4, Fig. 5). For both NPMR modelling scenarios, (1) percent foliar cover; (2) frequency; (3) change in cover; and (4) change in frequency were each modeled separately as the response variable. Predictor variables for the first scenario (geographic space) were Universal Transverse Mercator projection coordinates, and predictors for the second scenario (NMS ordination space) were NMS axes 1 and 2 [3] which were the axes that represented the greatest and second greatest variation occurring in species composition variability in our dataset. Axes 1 and 2 represented 64% and 23% of variation in the distance matrix respectively [3].

## 2 Experimental design, materials and methods

The ZPP was categorized into prairie and canyon lands and the canyon lands were excluded from this data [4]. The remaining prairie (∼5100 ha) was divided into quarter-quarter (0.25 × 0.25 miles or 16.2 ha) sections based on the US Public Land Survey System. 124 plots (∼1ha) were randomly located within each quarter-quarter section within the prairie using ArcMap [3]. A GEO-Explorer Trimble 3 handheld Global Positioning System was used to navigate to the selected plots. Three line-point intercept [1] transects (length = 50 m each), in a spoke design, radiating out from the center of the plot at 0°, 120°, and 240° relative to magnetic North were located within each plot [2,4]. Each transect was started 5 m from the center of the plot to exclude sampling in the very center of the plot which tends to get trampled by researchers during sampling. Species intercepts with transects were observed at 1 m increments, for a total of 150 points sampled (50 per transect) in each plot. For a given species, foliar cover (per plot) was calculated as the total number of hits for a given species divided by 150 multiplied by 100. Presence absence of dominant non-native grass species were also recorded within quadrats (0.4 × 0.4 m) spaced at 5 m increments along each transect line for a total possible frequency of 30 quadrats per plot. Frequency of occurrence for a particular species, in a plot, was calculated by dividing the number of quadrats the species of interest occurred in by the total of 30 and multiplying by 100. Plot sampling within each period was staggered one year, i.e., plots sampled in 2008 were resampled in 2015, and plots sampled in 2009 were resampled in 2016 to maintain a seven year time gap between re-samples for each plot. Plots were classified into three different plant community types (old fields, xeric prairie, and mesic prairie) based on species composition using cluster analysis and NMS ordination. Refer to [3,4] for interpretation and detailed discussion of results.

Change in abundance between the two time periods was calculated for each species as pairwise differences (plots were sample units) in percent foliar cover for the 30 dominant species and pairwise differences in frequency for dominant non-native grass species respectively for each of the three plant communities. Boxplots ordered by species rank abundance (with respect to the earlier time period) show distributions of pairwise differences in abundance of dominant plant species stratified by grassland community type [Fig. 1, Fig. 2, Fig. 3]. NPMR (HyperNiche 2.30 [5]) was used to generate 3-dimensional response surfaces for change in *Ventenata dubia* foliar cover and frequency within quadrats in both NMS ordination and geographic space (Fig. 4, Fig. 5). NPMR is a non-parametric regression method for fitting responses as a function of two or more predictors which are combined using a multiplicative weighting function [6]. NPMR automatically models interactions among predictors and has built in over-fitting protection consisting of a leave-one-out cross validation method during model fitting [6]. Cross validated *R*^2^ (*χ*R^2^) values were used to evaluate model fits and differ from the conventional *R*2 because it is based on the exclusion of each data point from the estimate of the response at that point [6]. Relative importance of each predictor to NPMR models were evaluated using sensitivity analysis [6]. Sensitivity is the ratio of the relative mean change in the response to the relative mean change in the predictor, i.e., a sensitivity (*S*) of 1.0 means that a 5% change in the predictor would result in, on average, a 5% change in the response [6]. We used a local mean estimator, Gaussian kernel smoother, automatic average minimum neighborhood size, with minimal backtracking and the Aggressive overfitting control setting in HyperNiche 2.30. All figures were created using the ggplot2 package [7] in R [8].

## Appendix A Supplementary data

Multimedia component 1 “Species_List.csv” contains a list of the 31 dominant species presented in this data paper. Columns include species symbol (USDA Plant Database); Species Scientific Name, Common Name, Family, Life form (F, Forb; G, Grass), Life Span (A, Annual; P, Perennial), and Nativity (N, Native to contiguous United States; NN, Non-native to contiguous United States). Multimedia component 1

Multimedia component 2 “Species_Matrix.csv” is the species matrix for both sampling periods (2008 & 2009 and 2015 & 2016). This species matrix includes sample units (plots) as rows, species as columns (species symbol; USDA Plant Database; https://plants.sc.egov.usda.gove/java/), and each cell represents the foliar cover estimate for that species in a given plot. Two extra columns, CommunityType (1, Old field community; 2, Mesic prairie community; 3, Xeric prairie community), and SamplingPeriod (1, 2008 & 2009; 2, 2015 & 2016). Multimedia component 2
